# Predictors of survival rate in patients with pancreatic cancer: A multi‐center analytical study in Iran

**DOI:** 10.1002/cnr2.1547

**Published:** 2021-09-07

**Authors:** Mansour Bahardoust, Mohammad Ali Abyazi, Sayed Ali Emami, Parmida Ghadimi, Mehrdad Khodabandeh, Farhad Mahmoudi, Ramin Hosseinzadeh, Mohammad Heiat, Shahram Agah

**Affiliations:** ^1^ Baqiyatallah Research Center for Gastroenterology and Liver Diseases Baqiyatallah University of Medical Sciences Tehran Iran; ^2^ Department of Epidemiology School of Public Health, Shahid Beheshti University of Medical Sciences Tehran Iran; ^3^ Heart Failure Research Center Isfahan Cardiovascular Research Institute, Cardiovascular Research Institute, Isfahan University of Medical Sciences Isfahan Iran; ^4^ Faculty of Medicine Iran University of Medical Sciences Tehran Iran; ^5^ Department of Physical Medicine and Rehabilitation Iran University of Medical Sciences Tehran Iran; ^6^ Medical Students Research Center Isfahan University of Medical Sciences Isfahan Iran; ^7^ Colorectal Research Center Iran University of Medical Sciences Tehran Iran

**Keywords:** Iran, Kaplan–Meier, pancreatic cancer, prognostic factors, survival rate

## Abstract

**Background:**

Pancreatic cancer (PC) is among the deadliest cancers of the gastrointestinal tract worldwide and a growing global health concern.

**Aim:**

This study was aimed to evaluate the survival rate and prognostic factors of survival in patients with PC.

**Methods:**

In this retrospective cohort study, the records of 556 patients with PC registered in the hospital cancer registration system from September 2007 to September 2020 were evaluated. In this regard, demographic data, tumor characteristics, received treatments, and patients' final status were analyzed. Kaplan–Meier and Cox's regression were used for univariate and multivariate analyses, respectively.

**Results:**

The 5‐year survival rate was found to be 4.3%. The median survival time was 12.4 ± 6.6 months. Univariate analysis showed that age, BMI (kg/m^2^), blood transfusions, differentiation, tumor stage, tumor size, number of involved lymph nodes, lymph node ratio (LNR), and type of treatment received were significantly associated with patient survival (*p* < .05). Multivariate Cox regression indicated that the age ≥60 years [Hazard Ratio (HR) = 1.25, 95% confidence interval (CI) = 1.03–1.49], BMI <18 (kg/m^2^; HR = 1.56, 95% CI = 1.13–2.14), poor differentiation (HR = 2.12, 95% CI = 1.75–2.49), tumor size >2.5 cm (HR = 4.61, 95% CI = 3.30–6.78), metastasis presence (HR = 1.97, 95% CI = 1.49–2.60), more than two involved lymph nodes (HR = 1.52, 95% CI = 1.31–1.77), LNR <0.2 (HR = 0.56, 95% CI = 0.36–0.77), and adjuvant therapy with surgery and chemotherapy (HR = 0.44, 95% CI = 0.28–0.61) are the most important prognostic factors of survival in patients with PC (*p* < .05).

**Conclusions:**

This study showed that the survival rate of patients with pancreatic cancer varies based on the characteristics of the tumor and the type of treatment received.

## 1 INTRODUCTION

Pancreatic cancer (PC) is among the deadliest cancers of the gastrointestinal tract and the eighth leading cause of cancer death with more than 250 000 annual deaths globally.^1^ It is estimated that PC would be the second leading cause of cancer deaths by 2030. The mortality rate of PC is higher in males than in females, and its rate increases with age.^2^, ^3^ PC has the worst prognosis among all cancers. It is the only cancer with an annual incidence higher than its prevalence.^4^ Iran ranks 11th among Asian countries regarding the mortality rate and incidence of PC. This cancer is the 12th leading cause of cancer death in Iran.^5^, ^6^ The survival of patients with PC depends on several factors such as the type of treatment received, the patient age at the time of diagnosis, the number of involved lymph nodes, metastasis, tumor size, and tumor stage.^7^, ^8^ The 5‐year survival rate of patients with PC has been reported below 5%, and most patients die within 6 months after diagnosis.^1^ Tumor removal by surgery is the only appropriate treatment to increase the survival rate of patients with PC. However, most patients are diagnosed in advanced stages of the disease with metastasis, and only 10–20% of patients refer for surgery at the appropriate time.^9^ The mean approximate survival time of patients with metastasis is 6 months, and the median survival time in advanced stages of the disease is reported to be 4–12 months.^2^, ^10^, ^11^

The survival period of patients diagnosed with PC has been the subject of numerous epidemiological studies in Iran.^12^, ^13^, ^14^ No study has been conducted in Iran to evaluate the survival predictors among patients with PC. On the other hand, it is necessary to evaluate patients and select the best treatment protocol. Accordingly, this study was designed to evaluate the survival rate and prognostic factors of survival in patients with PC for the first time in Iran.

## 2 MATERIALS AND METHODS

This retrospective study was approved by the Research Ethics Committee of Baqiyatallah University of Medical Sciences, Tehran, Iran (IR.BMSU.REC.1398.318). The informed consent of patients was obtained to use their medical information. The methods were carried out following the relevant guidelines and regulations.

The medical records of 556 patients with a confirmed PC diagnosis referred to two medical centers in Teheran from the beginning of September 2007 to September 2020 for cancer treatment (tumor removal or chemotherapy) were investigated. The inclusion criteria were definitive diagnosis of PC and patients with available records. Exclusion criteria were the lack of access to files and incomplete information, patients with concurrent cancers, and patients with a follow‐up period of fewer than 6 months. The patients with PC were diagnosed by a gastroenterologist and an oncologist based on endoscopic ultrasound and pathological findings. All researches during this study were according to the principles of the Helsinki Declaration.

The demographic information of patients (age, gender, family history of cancer, body mass index [BMI], alcohol consumption, and smoking) and the laboratory and clinical information (platelet counts, history of blood transfusion, time of diagnosis, time of death, type of treatment received, and patient death or survival) were extracted by referring to the patients' medical records in the cancer registration system in the hospital archives. All information was completed and collected by a researcher using a checklist by reading the cancer registration system file. Tumor characteristics, including tumor differentiation, stage, size, number of involved nodes, and presence of metastasis, were extracted from pathological and histological reports of patients.

From the Tumor‐Node‐Metastasis index (TNM), the tumor size (≤2.5 cm >2.5 cm), number of the lymph nodes involved (≤2 vs. >2), and metastasis (positive/negative) to other organs were used to classify the disease severity and estimate patient survival.^15^ The LNR was calculated by dividing the total number of lymph nodes harboring metastasis by the total number of nodes. Patients were then divided into four groups based on their LNRs. Patients with N1 disease in the node‐negative group (N0, LNR: 0) were subclassified into three groups using the cutoff values of 0.2 and 0.4) ≤0.2, 0.2–0.4, and >0.4). The mortality rate of patients was considered the main outcome of the study. The survival rate was defined as the time interval between the first definitive diagnosis of PC and death.

### 2.1 Statistical analysis

The collected data was analyzed with the help of SPSS 22 (IBM Inc., Chicago, IL). The Kaplan–Meier with log‐rank test was used to compare the distribution of the baseline variables, including demographic data (gender, age, and family history), clinical parameters, and tumor characteristics (platelet level, blood sugar, underlying diseases, tumor size, number of lymph nodes involved, metastasis, and the type of treatment received) and plot the survival curve after diagnosis. The survival rate was reported by the Kaplan–Meier curve based on different variables. The Cox regression univariate analysis with *p* < .10 was used to evaluate the effect of variables on patients' survival. Variables with *p* < .10 in the log‐rank test were entered into the Cox multivariate analysis with the backward selection method. The Cox proportional hazard model was used for multivariate analysis. Hazard ratios (HRs) and 95% confidence intervals (CIs) were calculated. A *p*‐value of less than .05 was considered statistically significant.

## 3 RESULTS

### 3.1 Demographic and clinical data

In total, the records of 556 patients were reviewed. Of this, 306 (55%) patients with PC were male, and 45% were female. The patients' age ranged from 32 to 81 years with a mean age of 61.6 ± 30.3 years. The mean BMI of patients was 20.16 ± 5.4 kg/m^2^, 338 (60.7%) patients had underlying diseases (diabetes, hypertension, and hyperlipidemia), and 216 patients (38.8%) had a history of receiving more than two units of blood. The mean platelet count was 146.6 ± 32.5, 224 (40.3%) patients had poor tumor differentiation, 254 patients (45.7%) had stage 2 tumors, and 384 (69.1%) had a tumor larger than 2.5 cm. The tumor had metastasized to distant organs in 188 (33.8%) patients, and more than two nodes were involved in 372 (66.9%) cases. The most common treatment methods were surgical treatment and adjuvant therapy with surgery and chemotherapy. Table 1 summarizes the distribution of demographic, laboratory, and pathological variables and tumor characteristics.

**TABLE 1:** Distribution of demographic, laboratory, pathological and tumor characteristics

Variables	Frequency (%)
Demographic data	
Age (mean ± SD)	61.6 ± 30.3
Sex	
Male	306 (55%)
Female	250 (45%)
Mean follow‐up (months)	12.7 ± 11.3
Median survival (months)	12.4 ± 6.6
BMI (kg/m^2^)	20.16 ± 5.4
Family history(positive)	11 (3.9%)
Alcohol consumption (positive)	104 (18.7%)
Comorbidity (positive)	338 (60.7%)
Smoker(history)	92 (16.5%)
Laboratory data	
Hemoglobin (g/dl)	13.52 ± 5.6
Platelet count × 10^3^ (g/dl)	146.6 ± 32.5
Blood transfusions	
≤2 units	202 (36.3%)
>2 units	216 (38.8%)
None	138 (24.9%)
Differentiation	
Poor	224 (40.3%)
Moderate	286 (51.4%)
Well	56 (8.3%)
Tumor characters	
Tumor stage	
I	54 (9.7%)
II	254 (45.7%)
III	194 (34.9%)
IV	54 (9.7%)
Tumor size (cm)	
≤2.5	172 (30.9%)
>2.5	384 (69.1%)
Metastasis presence	
Negative	306 (55%)
Positive	188 (33.8%)
A known	62 (11.2%)
Number of involved lymph node	
≤2	184 (33.1%)
>2	372 (66.9%)
Treatment type	
Surgery	196 (35.3%)
Chemotherapy	126 (22.7%)
Radiotherapy	38 (6.8%)
Surgery and chemotherapy	140 (25.2%)
Unknown	56 (10%)

### 3.2 Survival

**FIGURE 1:**
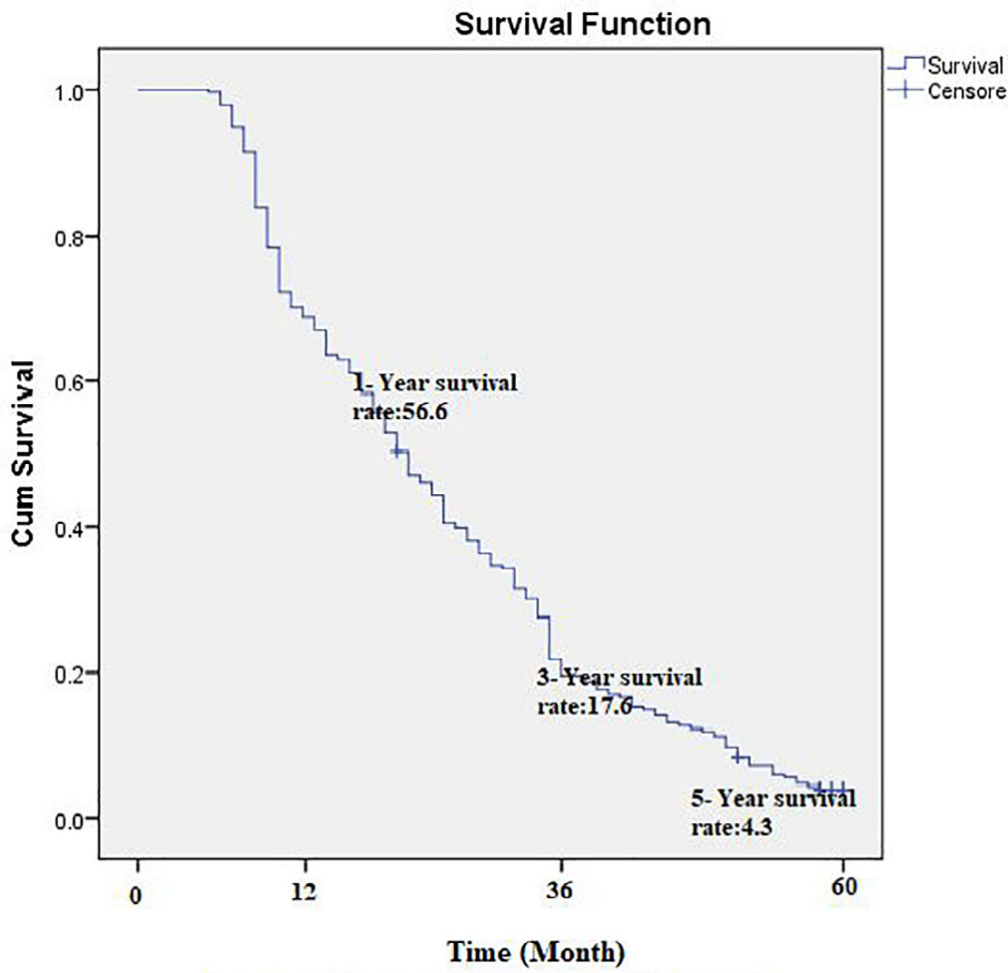
Kaplan–Meier curve, survival rate

The average follow‐up time was 12.7 ± 11.3 months, and the overall median survival time of patients was 12.4 ± 6.6 months. The one‐year survival rate of patients with PC was estimated at 56.6%. The 3‐ and 5‐year survival rates of patients were 17.6 and 4.3%, respectively (Figure 1).

**FIGURE 2:**
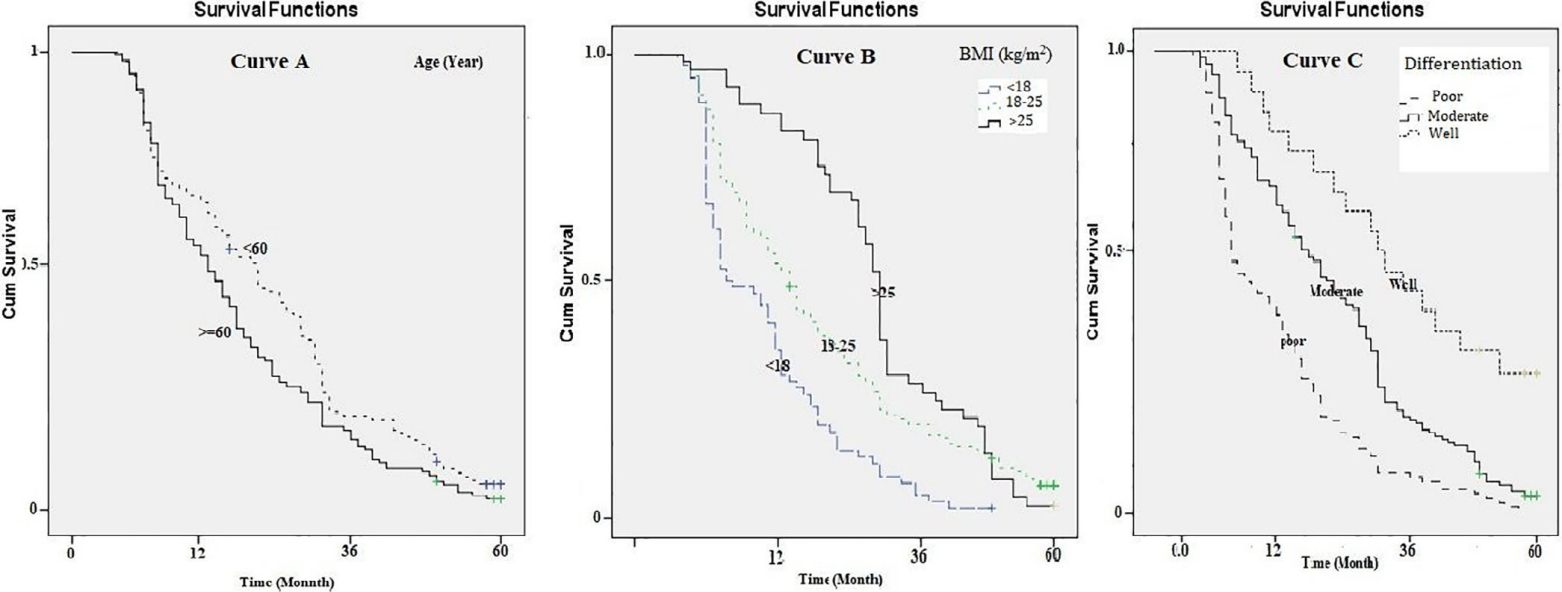
Five‐year survival rate (Kaplan–Meier) of patients with pancreatic cancer based on the age, BMI and histology characteristics of patients

The median survival of patients aged over and less than 60 years was, respectively, estimated at 10.5 and 16.1 months, statistically significant (Log‐rank Test chi^2^ = 5.03, *p* = .024; Figure 2: curve A). The median survival of patients with BMI <18 kg/m^2^ (7.5 months) was significantly lower than those with a BMI of 18–25 kg/m^2^ (Log‐rank Test chi^2^ = 25.37, *p* = .001; Figure 2: curve B). The median survival period was 8.9, 15.4, and 29.4 months for patients with poor, moderate, and well tumor differentiation, respectively. The median survival period of patients with poor tumor differentiation was significantly shorter than those with moderate and well‐differentiated tumors (Log‐rank Test chi^2^ = 29.54, *p* = .001; Figure 2: curve C; Table 2).

**TABLE 2:** Predictors of 5‐year survival rate using the method Kaplan–Meier and log‐rank test

Variable		Median survival (month)	Log‐rank Test	*p*‐Value
Age (year)	<60	16.1 ± 2.6	5.033	0.024
	≥60	10.5 ± 1.4		
Sex	male	13.7 ± 1.38	0.52	0.44
	female	14.8 ± 1.83		
BMI (kg/m^2^)	<18	7.5 ± 2.78	25.37	0.001
	18–25	14.7 ± 1.3		
	25>	19.4 ± 0.89		
Family history	Negative	14.5 ± 1.89	1.25	0.78
	Positive	13.8 ± 2.1		
	Unknown	14.3 ± 2.65		
Blood transfusions	≤2 units	14.3 ± 1.92	1.65	0.16
	>2 units	13.1 ± 2.9		
	None	14.9 ± 1.2		
Platelet count × 10^3^ (g/dl)	≤150 >150	13.2 ± 2.6 14.8 ± 2.1	2.84	0.11
Differentiation	Poor	8.9 ± 2.88	29.54	0.001
	Moderate	15.4 ± 2.1		
	Well	22 ± 0.56		
Tumor stage	I	31.2 ± 3.57	68.64	0.001
	II	19.1 ± 1.35		
	III	6.4 ± 2.45		
	IV	3.2 ± 0.87		
Tumor size (cm)	≤2.5	24.4 ± 3.56	52.89	0.001
	>2.5	9.9 ± 2.78		
Metastasis presence	Negative	19.6 ± 3.25	67.5	0.001
	Positive	7.6 ± 1.02		
	A known	11.8 ± 1.31		
Number of involved lymph node	≤2	23.3	96.5	0.001
	>2	10.1		
LNR	0	33.5 ± 4.2	48.6	0.001
	<0.2	18.6 ± 3.2		
	0.2–0.4	8.8 ± 2.1		
	>0.4	4.2 ± 1.2		
Treatment type	Surgery	15.1 ± 3.14	88.65	0.022
	Chemotherapy	12.7 ± 1.56		
	Radiotherapy	4.9 ± 0.78		
	Surgery and chemotherapy	17.3 ± 3.68		
	Unknown	13.4 ± 1.56		

Abbreviation: LNR, lymph node ratio.

**FIGURE 3:**
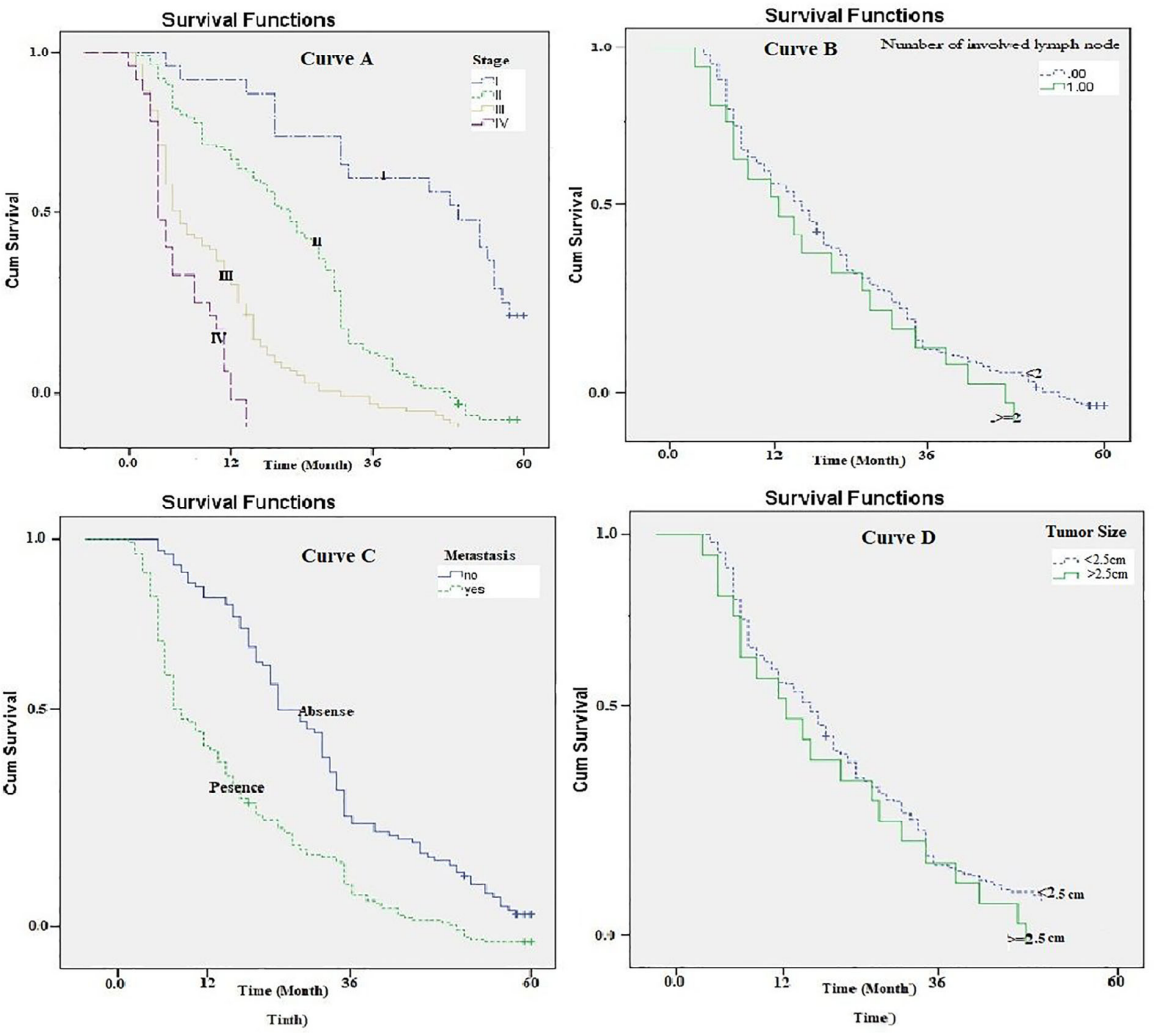
Survival rate (Kaplan–Meier) of patients with pancreatic cancer based on the tumor characteristics

The median survival period was significantly longer for patients with stage I tumor (Log‐rank Test chi^2^ = 68.64, *p* = .001; Figure 3: curve A). The median survival period of patients with a tumor size of less than or equal to 2.5 cm was significantly longer than those with a larger tumor size (Log‐rank Test chi^2^ = 52.89, *p* = .001; Figure 3: curve B).

The median survival period was significantly shorter for patients with metastases to other organs (Log‐rank Test chi^2^ = 67.5, *p* = .001; Figure 3: curve C). The median survival time (10.1 months) was significantly shorter for patients with more than two involved nodes than those with less involved nodes (23.3 months; Log‐rank Test chi^2^ = 96.5, *p* = .001; Figure 3: curve D).

**FIGURE 4:**
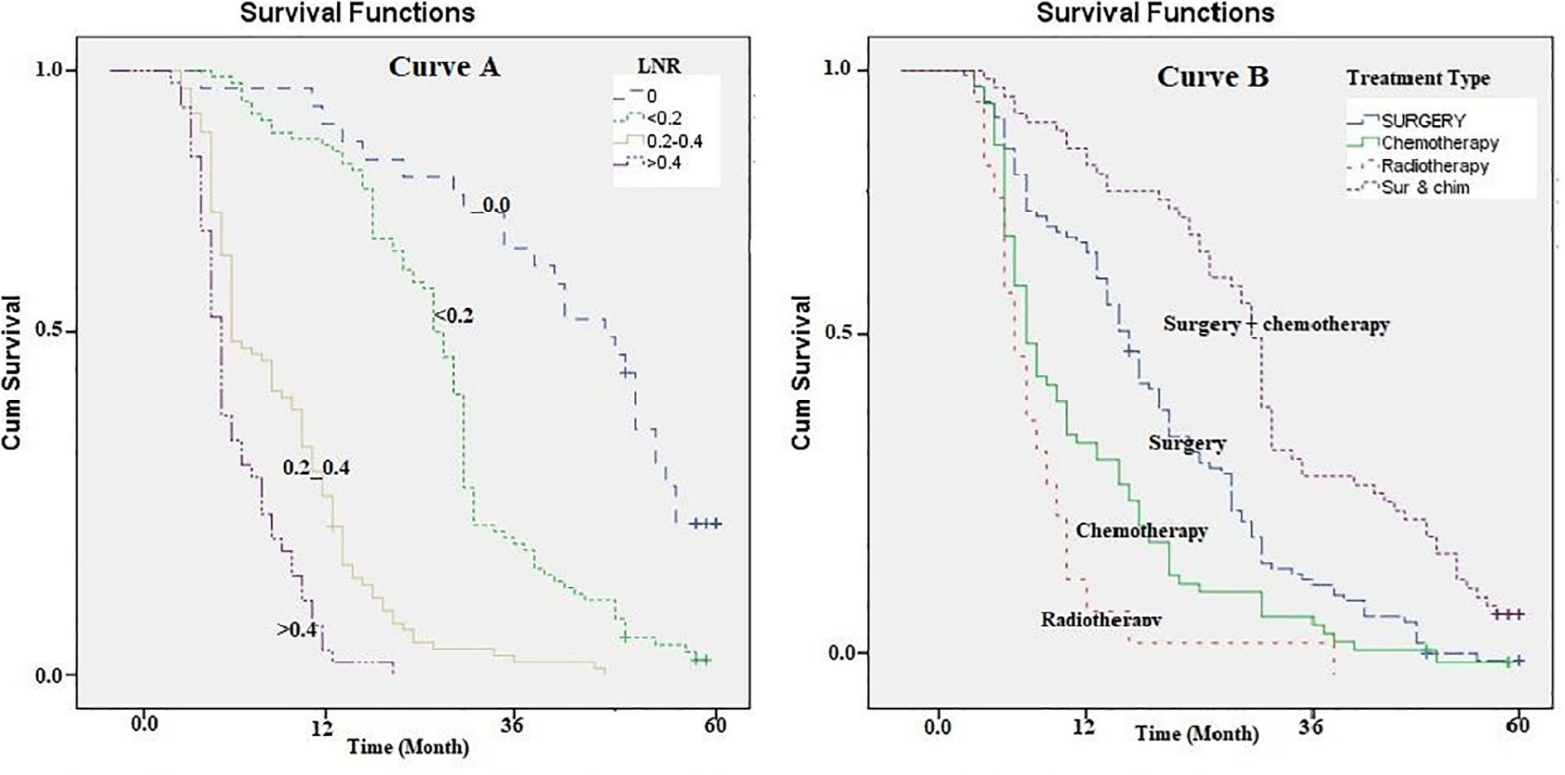
Five‐year survival rate (Kaplan–Meier) of patients with pancreatic cancer based on the lymph node ratio and type of treatment received

The median survival times for patients with LNRs of 0, ≤0.2, 0.2–0.4, and >0.4 were, respectively, 33.5, 18.6, 8.8, and 4.2, which were significantly different (Log‐rank Test chi^2^ = 48.6, *p* = .001; Figure 4: curve A). Finally, the survival rate was analyzed based on the treatments received by patients. The highest median survival rate was observed for those who underwent surgery and chemotherapy (17.3 months) simultaneously (Log‐rank Test chi^2^ = 88.65, *p* = .022; Figure 4: curve B, Table 2).

No significant difference was found between the patients' median survival period in terms of gender, family history of PC, alcohol use, blood transfusion, and platelet count (Table 2).

### 3.3 Univariate Cox regression

The univariate Cox regression analysis was used to determine the predictors of survival in patients with PC. According to the univariate Cox regression analysis results, age, BMI (kg/m^2^), platelet count, blood transfusions, tumor differentiation, tumor stage, tumor size, number of involved lymph nodes, LNR, and treatment method were significantly correlated with the survival of PC patients (*p* < .05; Table 3).

**TABLE 3:** Univariate analysis to identify variables associated with overall survival

Variable	HR	95% CI	*p*‐Value
Age; ≥60 vs. < 60	1.308	1.026_1.66	0.03
Sex; male vs. female	1.09	0.86_1.39	0.468
BMI (kg/m^2^)			
18–25	Reference		
<18	1.96	1.19_2.72	0.001
25>	0.81	0.59_1.04	0.075
Family history; Positive vs. negative	1.05	0.86_1.38	0.66
Blood transfusions; >2 vs. ≤2 units	1.09	1.01_1.22	0.048
Platelet count; >150 vs. ≤150 × 103 (g/dl)	0.89	0.78_0.99	0.038
Differentiation			
Poor	Reference		
Moderate	0.51	0.23_0.78	0.001
Well	0.21	0.11_0.32	0.001
Tumor stage			
I	Reference		
II	1.64	1.23_2.06	0.001
III	4.84	3.34_6.35	0.001
IV	8.64	6.22_10.87	0.001
Tumor size; >2.5 vs. ≤2.5 (cm)	4.93	3.49_6.98	0.001
Metastasis; presence vs. absence	2.12	1.65_2.74	0.001
Number of involved lymph node; >2 vs. ≤2	1.72	1.51_1.96	0.012
LNR			
0	Reference		
<0.2	1.8	1.54_2.01	0.001
0.2–0.4	3.8	2.34_5.27	0.023
>0.4	7.9	5.34_1.047	0.038
Treatment type			
Surgery	Reference		
Chemotherapy	1.2	1.01_1.42	0.032
Radiotherapy	3.5	2.24_4.75	0.001
Surgery and chemotherapy	0.86	0.75_0.97	0.024

Abbreviation: LNR, lymph node ratio.

### 3.4 Multivariate Cox regression

All variables significantly related to patients' survival rate in the univariate Cox regression analysis were analyzed by multivariate Cox regression. The multivariate analysis results showed that age ≥60 years, BMI <18 (kg/m^2^), poor tumor differentiation, tumor size >2.5, metastasis, more than two involved lymph nodes, and LNR >0.2 were significantly correlated with reduced survival of patients. In contrast, tumor stage I and adjuvant therapy (surgery plus chemotherapy) were significantly associated with increased survival of patients (*p* < .05; Table 4).

**TABLE 4:** Independent variables predictive of survival by multivariate analysis

Variable	HR	95% CI	*p* Value
Age; ≥60 vs. <60	1.25	1.03–1.49	0.031
BMI; <18 or >25 vs. 18–25 (kg/m^2^)	1.56	1.13–2.14	0.011
Differentiation; Poor vs. moderate/well	2.12	1.75–2.49	0.001
Tumor stage; I vs. II/III/IV	0.38	0.27–0.49	0.001
Tumor size;>2.5 vs. ≤2.5 (cm)	4.61	3.30–6.78	0.001
Metastasis; presence vs. absence	1.97	1.49–2.60	0.001
Number of involved lymph node; >2 vs. ≤2	1.52	1.31–1.77	0.001
LNR; <0.2 vs. 0.2–0.4/>0.4	0.56	0.36–0.77	0.013
Treatment; adjuvant therapy with surgery plus chemotherapy vs. other approaches	0.44	0.28–0.61	0.001

Abbreviations: CI, confidence interval; HR, hazards ratio; LNR, lymph node ratio.

## 4 DISCUSSION

Unlike most studies in which epidemiology, risk factors, and comparison of treatment methods for PC^6^, ^16^, ^17^, ^18^ have been considered, this study investigated the survival rate and predictors of survival among PC patients based on clinical characteristics and treatment methods. According to the Cox regression results in this study, age, BMI, tumor differentiation, tumor size, metastasis, number of involved lymph nodes, tumor stage, LNR, and the treatment type are related to the survival rate of PC patients. Adjuvant therapy was associated with increased patient survival. The 1‐, 3‐, and 5‐year survival rates of PC patients were estimated to be 56.6, 17.6, and 4.3%, respectively, consistent with those reported in the literature.^19^, ^20^

PC characteristics in Iran mirror the western countries so that males get affected more than females.^21^, ^22^ Similar to previous studies, our results also showed that males (55%) are more affected than females (45%). The literature reports the higher mortality rate of males than females.^23^, ^24^ However, no significant difference was found in patients' median survival period based on gender. The observed inconsistency could be attributed to the longer period in our study (13 years) compared to other Iranian studies such as Pourhoseingholi et al (9 years) and Salehi et al (4 years).^23^, ^24^ Moreover, this mismatch can root in the smaller volume of our sample than that in Pourhoseingholi et al and Salehi et al. It has been observed that the incidence of PC in Iranians peaks in the eighth decade of life.^21^, ^23^

The literature indicates an increase in the incidence and mortality of PC in different parts of Iran.^24^, ^25^, ^26^ Smoking, aging, and lifestyle changes are the most critical risk factors for PC in Iran. The novelty of this study was to define prognostic factors of survival among PC patients based on tumor characteristics and the type of treatment received. Our findings revealed that age ≥60 years, poor tumor differentiation, tumor size >2.5 cm, the presence of metastasis, the number of involved lymph nodes >2, LNR <0.2, and adjuvant therapy were the most important prognostic factors of survival in patients with PC.

Ilic et al reported a 5‐year survival rate of 2–9% for PC patients in different countries, consistent with our study.^16^ Kimura et al measured the survival rate of 147 patients with PC after pancreatectomy and found 3‐ and 5‐year survival rates of 18.4 and 12.2%, respectively.^19^ However, the 5‐year survival rate of patients in their study was higher than that in this study, presumably, due to their inclusion criteria, which were limited to only those who underwent pancreatectomy.

According to our results, age ≥60 years and 25 < BMI < 18 kg/m^2^ were significantly associated with decreased patient survival, consistent with Prashanth et al.^20^ In another study, Johnston et al found a significantly higher survival rate in younger patients.^27^ Their study demonstrated that patients were 13% more likely to die for each increasing decade of age due to the aggravation of other tumor features. Donghui Li et al observed a decrease in the survival rate of patients with a BMI more or less than normal,^18^, ^19^, ^20^, ^21^, ^22^, ^23^, ^24^, ^25^ which can be due to other underlying metabolic diseases in obese patients or weakness, anorexia, and poor health status in low‐weight patients.^28^ Therefore, BMI can be considered a risk factor for the reduced survival rate of PC patients. In general, few studies have been conducted on the correlation between BMI and PC survival rate.

Furthermore, Cox multivariate analysis results showed that tumor size >2.5 cm, metastasis, more than two involved lymph nodes, and higher tumor stage were significantly correlated with the reduced survival rate of PC patients. These findings are consistent with those reported in the literature.^19^, ^27^, ^29^ Based on our results, the median survival period was significantly longer in patients with tumor size >2.5 than those with tumor size <2.5 (9.9 vs. 24.4 months). Johnston et al found significantly lower survival rates in patients with tumor size larger than 2 cm,^27^ consistent with our results. In another study, Fortner et al found a significantly higher 5‐year survival rate in patients with tumor size <2.5 cm than those with tumor size >2.5 cm (33 vs. 12%),^30^ consistent with our results. Kimura et al evaluated the survival rate and clinical and pathological characteristics of 147 PC patients in a retrospective study from 1988 to 2012.^19^ According to their results, the average survival rate was estimated to be 14.4 months. They also showed that the presence of metastasis was significantly associated with a reduced patient survival rate. The patient's survival in the advanced stages of the disease was also less than that in the early stages.^19^ These results were consistent with our results, which reported a significantly shorter median survival period for patients with metastasis (7.6 months) than those without metastasis. In another study, Waraya et al introduced metastasis as a predictor of the reduced survival rate of PC patients.^31^

The multivariate Cox analysis implied that more than two involved lymph nodes were associated with a decreased survival rate of PC patients. Similar to the results of our study, Johnston et al,^27^ Waraya et al,^31^ and Pawlik et al^32^ reported more than two involved lymph nodes as a risk factor of decreased survival in PC patients. In agreement with our results, they also found a lower survival rate of patients in the advanced stages of the disease than in the early stages.

The multivariate Cox analysis results showed that adjuvant therapy (surgery plus chemotherapy), contrary to other treatments, was significantly associated with an increase in the survival rate (HR: 0.44) so that the median survival period in patients who simultaneously underwent surgery and chemotherapy (17.3 months) was significantly longer than other treatments. The median survival period was also significantly longer in patients treated with surgery alone (15.1 months) than those treated with chemotherapy (12.7 months) or radiotherapy (4.9 months). According to Mohamad et al,^33^ surgery (HR: 0.4) versus chemotherapy (HR: 0.53) and radiotherapy (HR: 0.66) were significantly associated with an increased survival rate of PC patients, consistent with our results. Unlike our study, they did not consider the role of combined therapy in their study. Johnston et al^27^ and Waraya et al^31^ showed that adjuvant therapy was significantly associated with increased survival of PC patients.

According to the results, the median survival period for patients with poor tumor differentiation was significantly shorter than those with moderate differentiation (8.9 vs. 15.4 months), consistent with those reported in the literature.^31^, ^34^ Consistent with our study, Waraya et al^31^ reported a significantly shorter median survival period for patients with poor tumor differentiation (11.4 months) than those with moderate tumor differentiation (21.1 months). According to the Cox multivariate analysis results, LNR >0.2 was significantly associated with the survival rate (HR: 0.56). The median survival period for patients with LNR >0.2 (8.8 months) was significantly shorter than those with LNR <0.2 (18.6 months).

Pawlik et al. evaluated the role of LNR in the survival of PC patients^32^ and estimated a median survival period of 21.7 and 15.3 months, respectively, for patients with LNR <0.2 and LNR >0.2, confirming our results. In general, limited studies on LNR and PC necessitate further investigations.

### 4.1 Strengths and limitations

Our study had weaknesses that should be noted. The most important weakness was the retrospective design and the use of patient records, which may be lost due to the lack of access to some fundamental data such as serum CA19‐9 levels in determining the prognostic factors of PC. Another limitation was the short follow‐up period due to the disease fatality or the late diagnosis of PC patients. The sample volume of 556 patients is not very large, and further studies with bigger sample sizes are needed to confidently generalize the results of this study. The main strength of our research was the multi‐center study design and the lack of missing data.

## 5 CONCLUSION

The results showed that aging, abnormal BMI, poor tumor differentiation, larger tumor size, metastasis, more involved lymph nodes, and higher LNR were the most important risk factors for the survival of PC patients. At the same time, combination therapy with surgery plus chemotherapy was significantly associated with an increased survival rate of PC patients. These results are essential in estimating the survival rate of PC patients and choosing the best treatment protocol for such patients.

## CONFLICT OF INTEREST

The authors declare no conflict of interest.

## AUTHOR CONTRIBUTIONS

*Conceptualization, Data Curation, Formal Analysis, Methodology, Supervision, Writing—Original Draft, Writing—Review and Editing*, M.B.; *Conceptualization, Writing—Original Draft, Writing—Review and Editing*, M.A.A.; *Data Curation, Investigation, Writing—Review and Editing*, S.A.E.; *Data Curation, Writing—Review and Editing*, P.G.; *Data Curation, Writing—Original Draft*, M.K.; Writing—Review and Editing, F.M.; Data Curation, Writing—Original Draft, R.H.; Conceptualization, Supervision, Writing—Original Draft, S.A.

## ETHICAL STATEMENT

This study was approved by the Vice Chancellor at the Research and Ethics Committee for Research of Baqiyatallah University of Medical Sciences, Tehran, Iran with number code. (IR.BMSU.REC.1398.318). The research team of this study adhered to the ethical principles of the Helsinki Convention regarding clinical studies in all stages of the present study. This study involved a retrospective review of medical records, the requirement for informed consent was waived by the ethic committee of Baqiyatallah University of Medical Sciences.

## Data Availability

The datasets used and analysed during the current study are available from the corresponding author on reasonable request.
